# Palladinized graphene oxide-MOF induced coupling of Volmer and Heyrovsky mechanisms, for the amplification of the electrocatalytic efficiency of hydrogen evolution reaction

**DOI:** 10.1038/s41598-021-96536-9

**Published:** 2021-08-26

**Authors:** Mogwasha D. Makhafola, Kwena D. Modibane, Kabelo E. Ramohlola, Thabiso C. Maponya, Mpitloane J. Hato, Katlego Makgopa, Emmanuel I. Iwuoha

**Affiliations:** 1 Nanotechnology Research Lab, Department of Chemistry, School of Physical and Mineral Sciences, University of Limpopo (Turfloop), Sovenga, 0727 Polokwane South Africa; 2 Department of Chemistry, Faculty of Science, Tshwane University of Technology (Acardia Campus), Pretoria, 0001 South Africa; 3 SensorLab, Chemistry Department, University of the Western Cape, Bellville, Cape Town, 7535 South Africa

**Keywords:** Chemistry, Energy science and technology, Materials science

## Abstract

In this study, a nanocomposite of palladium supported graphene oxide (GO)/metal–organic framework (MOF) was prepared using electroless deposition of Pd on GO followed by impregnation method of Pd@GO and MOF. The prepared materials were characterized with various analytical techniques and their applications as HER electrocatalysts were evaluated using cyclic voltammetry (CV), Tafel plots, and turn over frequencies (TOFs). The HER results showed a radical increment of H_2_ production in the nanocomposite through the Volmer reaction together with Heyrovsky or Tafel mechanism. This disclosed that the addition of Pd@GO/MOF in the electrolytic system possessed better catalytic characteristics with enhanced current density which may open a new way for hydrogen production and storage via HER.

## Introduction

The extreme reliance on fossil fuels for energy is escalating the global concern about air pollution and energy disaster^1^. Currently, there is an increasing courtesy on the development of sustainable and clean energy sources^2–4^. Hydrogen from water splitting is recognised as a potential energy carrier^5–7^. This is due to advantages such as recyclability, free pollution, and high energy efficiency as compared to gasoline-based fuels^5,7^. There are many ways for water splitting technology through hydrogen evolution reaction (HER), such as photoelectrochemical, photocatalytic, electrochemical, thermochemical, and photobiological methods^2,5^. Among many HER methods, eco-friendly and high purity of hydrogen can be obtained by an electrochemical technique which is considered as one of the most promising technologies for massive hydrogen production through hydrogen evolution reaction^7^. It was seen in the HER mechanism that Tafel analysis is used to get a better understanding of hydrogen production^5,6^. It was demonstrated that in an acid medium, the HER pathway could proceed through three main mechanisms^7,8^. The first mechanism involves the adsorption of hydrogen ions on the surface of the electrode, leading to the formation of the composite hydride in the hydrogen storage process as the most probable intermediate and proposed to be the Volmer mechanism^5–8^. After the hydride, the hydride intermediate easily undergoes a protonation or interacts with one another to form an H_2_ molecule through a Heyrovsky or Tafel reaction^8^. However, electrochemical water splitting hydrogen generation mainly depends on the cathode material used for HER to occur at the cathode electrode^6,8,9^. Even though platinum-based materials have shown to be the efficient electrocatalysts for HER, low earth-abundance and cost implications restrict their widespread uses^10^. Accordingly, a replacement of these platinum-based materials is paramount important^10–14^. Non-noble catalysts have been shown as alternative materials because of their exceptional electrical conductivity and outstanding durability^12–14^, yet, their limited active sites limit their HER performance^6,15^.

Metal–organic frameworks (MOFs) have been well-known as alternative catalysts for HER due to their large surface, low density, and controllable 3D structure^16,17^. There are few reports that showed the use of these materials as electro/photocatalyst in water splitting^14,18,19^. Nevertheless, MOFs displayed low electrochemical hydrogen production due to poor conductivity caused by their organic linker^14^. Therefore, there is a need to improve the physical and electrochemical properties of MOFs. Recently, Ramohlola et al.^20–22^ presented polymer-based metal–organic framework composites with an enhancement in electrocatalytic H_2_ evolution and higher Tafel slope in H_2_SO_4_ due to an improved electron density of the polymer by addition of MOF. Moreover, it has been reported that modification of MOF surface with a carbon-based material such as graphene oxide (GO) improves the structural properties of MOF^23–27^ due to GO’s rich functional oxygen groups and conjugate network of graphitic structure. These structural properties of GO enhance the electrical conductivity and the dispersive forces when incorporating it into other materials to form composites^23,24^. For example, a composite of cobalt oxide (Co_3_O_4_)-decorated reduced graphene oxide (rGO)-based nanoelectrodes showed to have good electrochemical performance for water splitting reactions in an alkaline medium^3^. Sapner et al*.*^11^ reported a functionalisation of GO with terminal nitrogen-containing groups (l-lysine) which resulted in efficient and stable electrocatalytic activity. The HER activities of rhodium nanoparticles decorated on graphene oxide (Rh–GO) were reported by Narwade et al.^7^. They showed that Rh–GO composite possessed an improved electrochemical performance for water splitting reactions with a small overpotential of 2 mV for the HER at a current density of 10 mA cm^−2^ and Tafel slope of 10 mV dec^–1^. The nickel/nickel oxide on reduced graphene oxide (Ni/NiO@rGO) composite demonstrated to have an overpotential of 582 mV at a current density at 10 mA cm^−2^ with a Tafel slope of 63 mV dec^−1^ obtained in 0.5 M H_2_SO_4_ towards HER^12^. The establishment of GO/MOF composite was demonstrated by Petit and Bandosz^25,26^ which was formed through connections between metallic cores of MOF and oxygen groups of GO. It was seen that the formation of GO/MOF possessed a synergetic effect which was responsible for the improvement of hydrogen uptake^26,27^. In relation to this, Monama et al.^28^ introduced a porphyrin (phthalocyanine) on MOF using the impregnation method, followed by Pd electroless deposition. The resultant composite showed an enhanced HER catalytic efficiency. In this work, we present the dispersion of the Pd onto the GO surface and then later incorporate it with MOF, to increase the dissociation ability of the metal and the quantity of hydrogen to be adsorbed in HER. Palladium, as compared to Pt, is of very low cost and has a high affinity towards hydrogen^29^. The structure and morphology of the composite were characterised by various analytical techniques, and its electrochemical hydrogen production and storage performance was compared with the blank electrode and MOF and investigated through HER studies.

## Materials and methods

### Materials

Trimesic acid (H_3_BTC), copper nitrate trihydrate (Cu(NO_3_)_2_.3H_2_O), graphite powder, tetrabutylammonium perchlorate (TBAP), and sodium nitrate (NaNO_3_) were procured from Sigma Aldrich, South Africa. Dimethylformamide (DMF), palladium chloride (PdCl_2_), ammonium solution (NH_4_OH), ammonium chloride (NH_4_Cl), dimethylsulfoxide (DMSO), phosphoric acid (H_3_PO_4_), and sulphuric acid (H_2_SO_4_) were purchased from Rochelle chemicals, South Africa. Hydrochloric acid (HCl) and potassium permanganate (KMnO_4_) were acquired from SAARCHEM, South Africa. Hydrogen peroxide (H_2_O_2_) was purchased from Moncon, South Africa. The standard solutions were prepared from H_2_SO_4_ in DMSO with 0.1 mol L^−1^ TBAP as an electrolyte solution at 22 ± 2 °C controlled by the thermostat.

### Preparation of MOF and Pd@GO/MOF composite

MOF was prepared by the following hydrothermal procedure^30^. Concisely, 2.5 mmol (0.525 g) of H_3_BTC dissolved in 10 mL of ethanol and then mixed with 10 mL solution of 4.5 mmol (1.087 g) of Cu(NO_3_)_2_.3H_2_O and distilled water. The mixture was stirred for 30 min and then transferred to a 23 mL Teflon stainless-steel autoclave and sealed to react for 36 h at 120 °C in the thermostatic drying oven. After, the resulted product was filtered, washed with ethanol three times, and then dried at 50 °C overnight. GO; and Pd@GO and Pd@MOF were prepared using a modified Hummers approach^31^ and electroless Pd deposition method^28^, respectively.

Pd@GO/MOF composite was synthesised through the impregnation method^28^ of directly mixing Pd@GO with MOF. Briefly, 0.1 g of as-dehydrated MOF (at 150 °C for 1 h) was dispersed in 10 mL DMF. In a separate beaker, 0.1 g of Pd@GO (50 wt.% of Pd@GO loading in the composite) was dispersed in 1.4 mL DMF. The two mixtures were mixed for 24 h at 50 °C. The mixture was then filtered to obtain the desired product which was washed several times with ethanol. The resulted clean product was dried in an oven at 50 °C for 12 h.

### Materials characterization

Spectrum II spectrometer (PerkinElmer, South Africa) was used to record FTIR spectra from 400 and 4000 cm^−1^ at room temperature. The structural phases of the materials were determined using XRD (Philips PW 1830, Industrial Analytical (Pty) Ltd (South Africa), CuKα radiation, λ = 1.5406 Å). Thermal stability was evaluated using a thermogravimetric analyser (STA 4000, PerkinElmer, South Africa). The samples were heated from 30 to 500 °C at a rate of 20 °C min^−1^. Auriga field emission scanning electron microscope/energy dispersive spectroscopy (FESEM/EDS) from Carl Zeiss (Pty) Limited, South Africa, was used for morphological characterizations and elemental analysis. FEI Tecnai G^2^ 20 transmission electron microscopy together with energy dispersive x-ray spectroscopy (TEM/EDX) and high-resolution transmission electron microscopy together with selected area electron diffraction (HRTEM/SAED) from FEI Company (America) were used to study the internal morphology at 200 kV accelerating voltage with camera length of 100 mm. X-ray photoelectron spectroscopy (XPS) analysis was conducted in a Thermo Scientific ESCALAB 250Xi (Waltham, Massachusetts, U.S) with a monochromatic Al K_α_ (1487 eV) x-ray beam. The calibration or peak shift analysis was done using the valence band spectra instead of the carbon peak shift since carbon was monitored as part of the analysis. EPSILON electrochemical workstation (BioAnalytical Systems Incorporated (BASi), West Lafayette, IN, USA) was used for electrochemical characterisation and studies using gold (3 mm diameter, 0.071 cm^2^ area), Pt, and Ag/AgCl electrodes as a working electrode, counter electrode, and reference electrodes, respectively. Scanning of the MOF, Pd@MOF, and Pd@GO/MOF composite with the concentration of ~ 2.0 × 10^−4^ mol L^−1^ in 25 mL of 0.1 mol L^−1^ TBAP/DMSO electrolyte was from − 2.0 to 1.25 V at a scan rate of 0.02–0.10 V s^−1^. HER studies were done by varying the concentration of H_2_SO_4_ as H_2_ source and ~ 2.0 × 10^−4^ mol L^−1^ of MOF, Pd@MOF, and Pd@GO/MOF nanocomposite as electrocatalysts. The electrochemical impedance spectroscopy measurements for MOF, GO, Pd@GO, Pd@MOF, and Pd@GO/MOF composite were performed with the frequency ranging from 5.0 × 10^5^–0.7 Hz in 0.500 mol L^−1^ electrolyte at open circuit potential.

## Results and discussion

### Structural characterization

**Figure 1:**
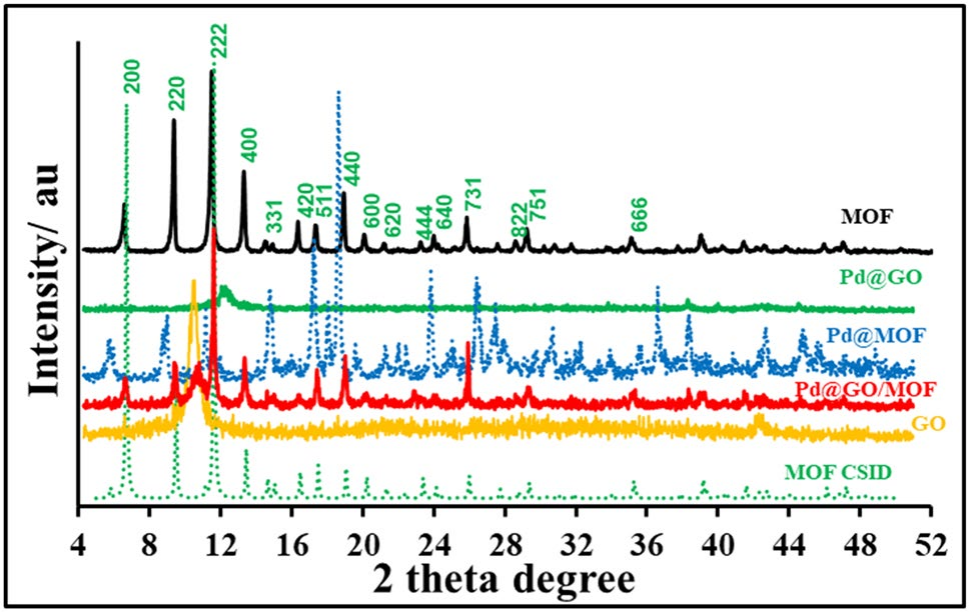
XRD patterns of MOF, GO, Pd@GO, Pd@MOF, and Pd@GO/MOF composite with reference to MOF CSID.

The XRD patterns of MOF, GO, Pd@MOF, Pd@GO, and Pd@GO/MOF nanocomposite are presented in Fig. 1. The pattern of MOF (Fig. 1) is characterized by an intense peak at 2 $\theta$= 12°, which is indexed as the (222) lattice plane with an interplanar d spacing of 7.68 Å (estimated using Bragg’s formula: d = (nλ)/(2sinθ), where d = interplanar spacing, n = positive integer, λ = wavelength and θ = scattering angle), typical for HKUST-1 type of MOF and is in agreement with MOF crystal structure information data (CSID)^30^. On the other hand, there was a reduction and shift of peak intensity of MOF phases with the introduction of Pd and the hkl (222) lattice plane with an interplanar d spacing of 7.64 Å. For GO, the pattern is observed to have one broad peak at 2 $\theta$=10.5° (d = 6.70 Å), corresponding to GO (002) crystal face^31,32^. However, it is observed that after the introduction of palladium, there is a shift of this mentioned peak to the right of the pattern, indicating the change in the environment of the oxygen-containing groups as palladium is incorporated on GO (Fig. 1). Characteristic peaks of palladium in Pd@GO are observed at 2 $\theta$ of 38 and 45° similar to the one observed on Pd@MOF^28^. It was reported that Pd coated materials have two reflection indexes of (111) and (200) at 2 $\theta$ values of 39 and 45°, respectively, as an indicator of Pd cubic phase^28^. The final composite Pd@GO/MOF consists of phases from both GO and MOF but with decreased and shifted intensities. The hkl indexes (002) and (222), of GO and MOF phases were calculated to have the interplanar d spacing of 8.73 and 7.64 Å, respectively. The palladium peaks are observed with low intensities as an indication of the palladium presence in the nanocomposite.

**Figure 2:**
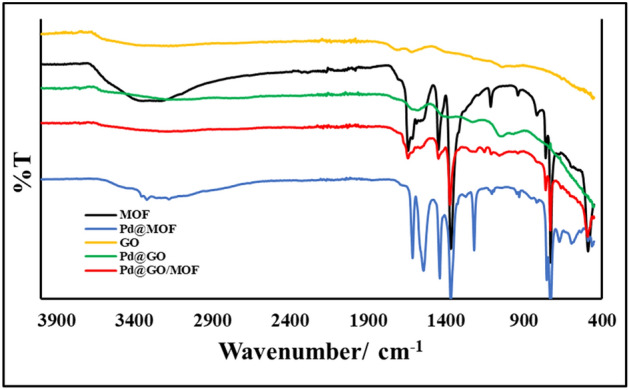
FTIR spectra of MOF, GO, Pd@GO, Pd@MOF, and Pd@GO/MOF composite.

Figure 2 displays the FTIR spectra of MOF, GO, Pd@GO, Pd@MOFand Pd@GO/MOF. The lattice vibrations of as-prepared MOF are in agreement with the literature^33^. In the spectrum of MOF, the coordination of the carboxylate group in the organic linker was observed at ~ 1300 cm^−1^^33^. The bands at ~ 1645 and 1450 cm^−1^ in the MOF spectrum also relate to the asymmetric and symmetric stretching vibrations of the C = O groups in BTC^28,33^. Lastly in the spectrum of MOF, a vibrational mode directly involving the Cu centre and the organic ligand (Cu–O) was observed at ~ 530 cm^−1^^20^. The spectrum of GO was reported elsewhere^34^ and possessed C–O and C = O vibrations at ~ 1060 and 1735 cm^−1^ from carboxyl and/or carbonyl groups^34^. Notably, the introduction of palladium onto GO and MOFreveals different interactions. The presence of Pd in Pd@GO is seen by the reduction in band intensities as compared to the GO spectrum^31^. Whereas in Pd@MOF, a new band was observed at ~ 1200 cm^−1^, which were corresponding to the characteristic peaks of Pd nanoparticles^4^.

The spectrum of Pd@GO/MOF shows the presence of MOF and Pd@GO functional group as an indication of the Pd supported composite formation.

**Figure 3:**
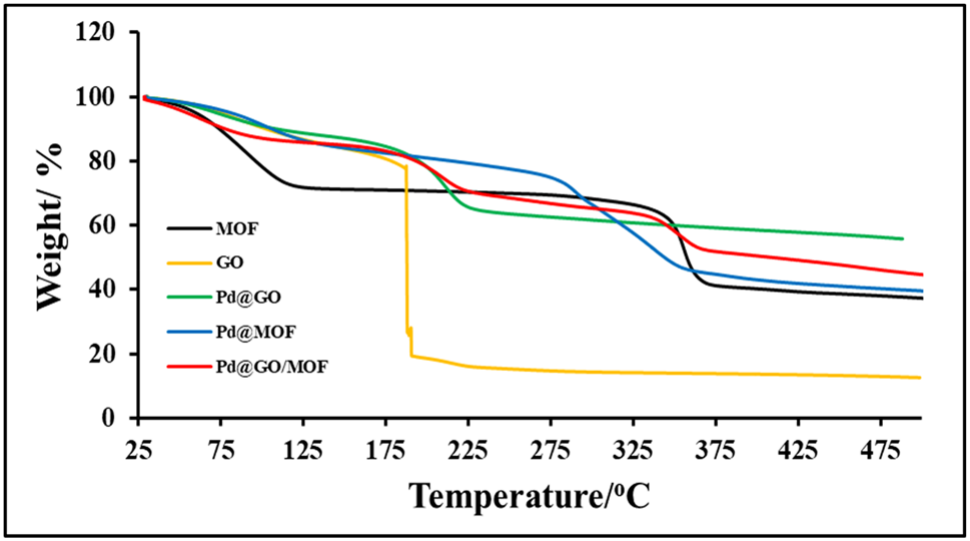
TGA analysis of MOF, GO, Pd@GO, Pd@MOF, and Pd@GO/MOF composite.

The thermal gravimetric analyses of the prepared materials are shown in Fig. 3. MOF is thermally stable up to 370 °C. The dehydration of MOF occurs at 100–125 °C and the framework's collapse at 350–370 °C^22^. Graphene oxide was reported to be thermally stable up to 180 °C, this collapse is accredited to the decomposition of oxygen-containing compounds^31,35^. Upon addition Pd on the surface of GO and MOF, the stability was enhanced as presented in Fig. 3 owing to the Pd interacting with GO and MOF, and hence limiting/reducing the loss in those functional groups. The addition of Pd@GO on the MOF surface displayed an improvement of stability as related to MOF and Pd@GO. Furthermore, the transitions of both MOF and GO are observed in the final composite, which confirms the incorporation between the two materials.

**Figure 4:**
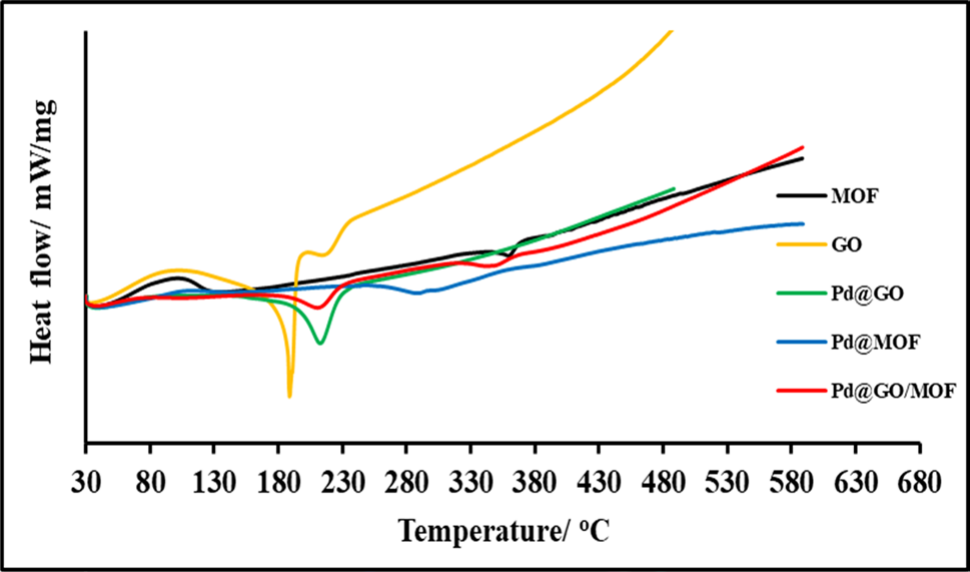
DSC analysis of MOF, GO, Pd@GO, Pd@MOF, and Pd@GO/MOF composite.

The Differential scanning calorimetry (DSC) results (Fig. 4) correlates well with the TGA analysis. There is an exothermic peak at 100–125 °C in MOF, which is due to the heat released when the dehydration process occurs^22^. Moreover, an endothermic peak at 350–370 °C is due to the absorption of heat as the frameworks of the MOF material collapses. With the introduction of palladium onto the MOF materials, there is an enhancement in thermal stability of the frameworks^28,31^. The Pd@GO shows an endothermic peak at 210 °C which is similar to the one of GO, due to the absorption of heat as material loses all the functional groups^31,35^. The final composite shows peaks corresponding to the loss of the two-parent materials (GO and MOF). This also confirms the formation of the composite Pd@GO/MOF.

### Morphological characterization

**Figure 5:**
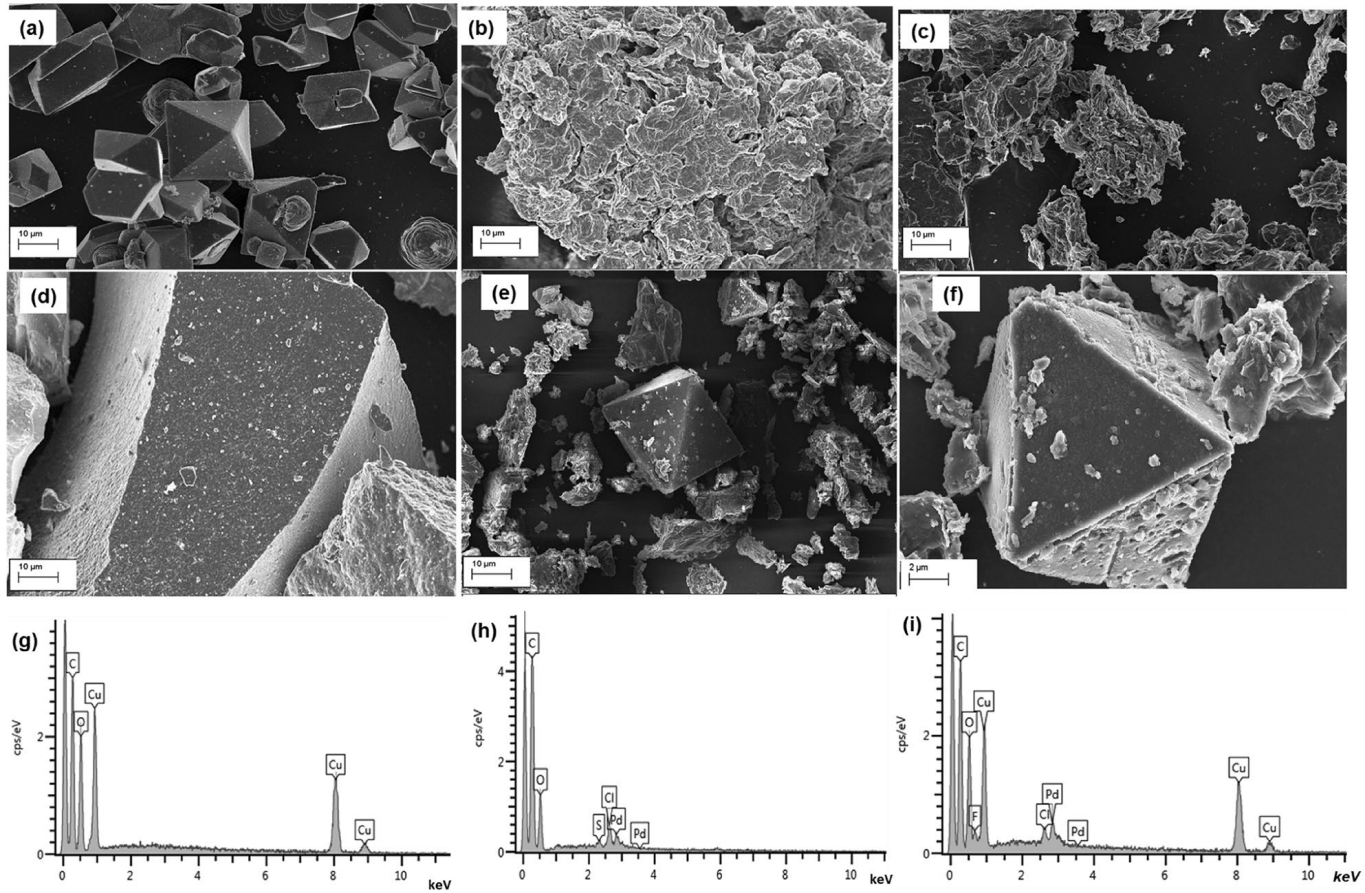
FE-SEM image of (**a**) MOF, (**b**) GO, (**c**) Pd@GO, (**d**) Pd@MOF, and Pd@GO/MOF composite at (**e**) low and (**f**) high magnification on the crystal structure to view the surface of the crystal; and EDS spectrum of (**g**) MOF, (**h**) Pd@GO, (**i**) Pd@GO/MOF composite*.*

The SEM images (Fig. 5a–f) were used to further provide the structural morphology and microstructure of MOF, GO, Pd@GO, Pd@MOF, and Pd@GO/MOF composite. As presented in Fig. 5a, MOF demonstrates the characteristic irregular shape of octahedral crystals^22,28^. The inset in Fig. 5a shows that MOF has a smooth surface. On the other hand, the EDS spectrum of MOF (Fig. 5g) discloses the amount of carbon, oxygen, and copper atoms present in the framework, and elemental analyses are given in Table 1. From the SEM image of Pd@GO crystals (Fig. 5c), it is clear to see well-dispersed nanoparticles of Pd on the GO surface without aggregation as compared to the SEM image of GO (Fig. 5b)^31^ This was further confirmed by the EDS spectrum of Pd@GO (Fig. 5h) also showing the presence of Pd atoms which are dispersed through the GO sheets and Pd atomic percentage is given in Table 1. It can be seen that the Pd nanoparticles were uniformly distributed on the surface of MOF (Fig. 5d). From the image of the Pd@GO/MOF composite, the rough surface due to the introduction of Pd@GO on MOF is observed (Fig. 5e). The high magnification image in Fig. 5f for Pd@GO/MOF composite displays the interconnecting of particles on the surfaces of the MOF structure. This remark is ascribed to the morphology of Pd@GO on the MOF structure as compared to neat MOF. EDS (Fig. 5i) showed the presence of Pd nanoparticles in the composite at 3.23 atomic percentage (Table 1).

**Table 1:** Elemental analysis of MOF, Pd@GO, and Pd@GO/MOF composite using EDS and EDX.

Technique	Material	Carbon at.%	Oxygen at.%	Copper at.%	Palladium at.%
EDS	MOF	66.89	26.01	7.1	–
	Pd@GO	70.23	23.41	–	3.98
	Pd@GO/MOF	54.84	32.26	8.06	3.23
EDX	MOF	37.84	8.11	54.05	–
	Pd@GO	81.08	16.22	–	2.70
	Pd@GO/MOF	59.78	16.30	21.74	2.17

**Figure 6:**
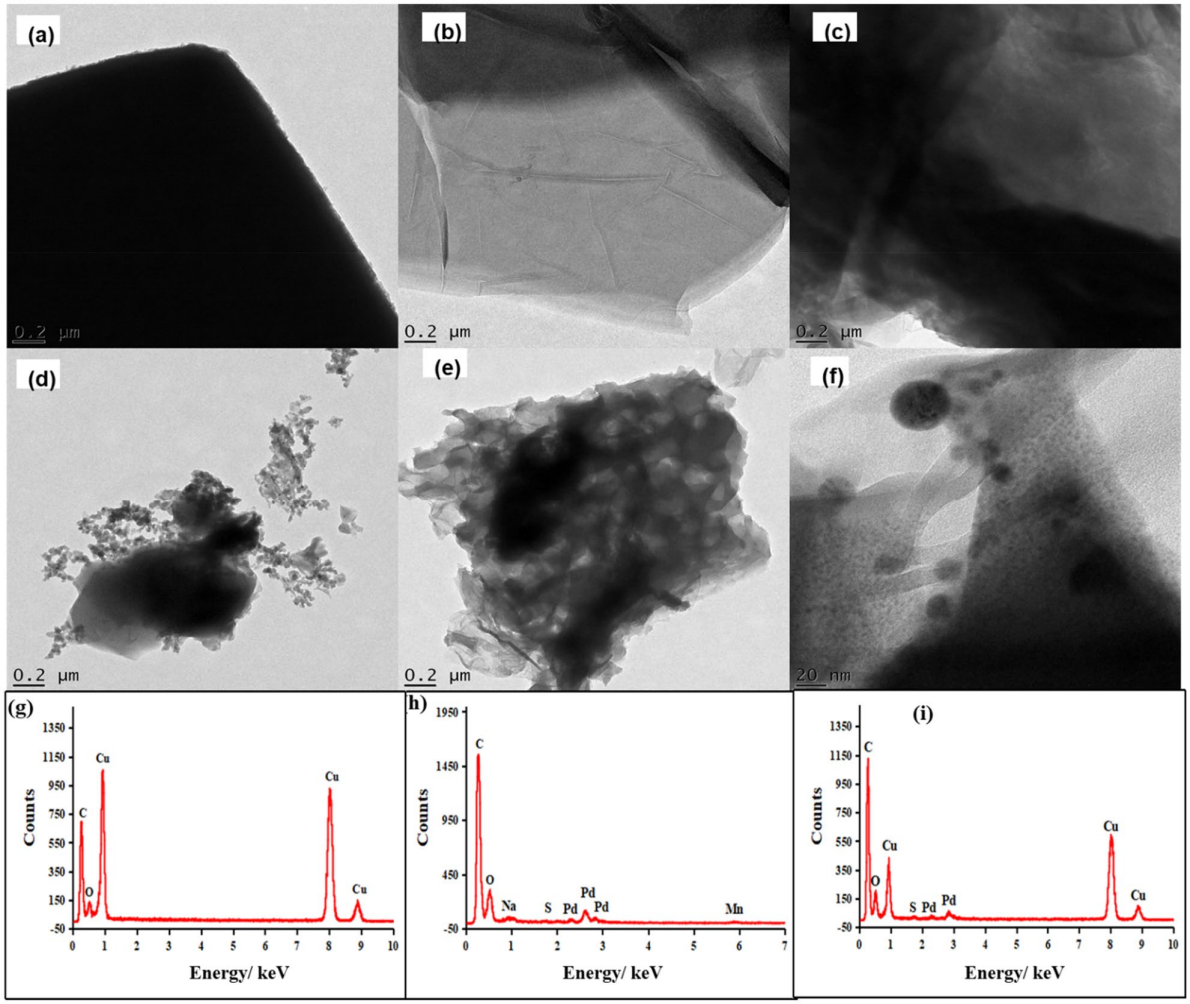
TEM image of (**a**) MOF, (**b**) GO, (**c**) Pd@GO, (**d**) Pd@MOF, and Pd@GO/MOF composite at (**e**) low and (**f**) high magnification on the crystal structure to view the surface of the crystal; and EDS spectrum of (**g**) MOF, (**h**) Pd@GO, (**i**) Pd@GO/MOF composite*.*

**Figure 7:**
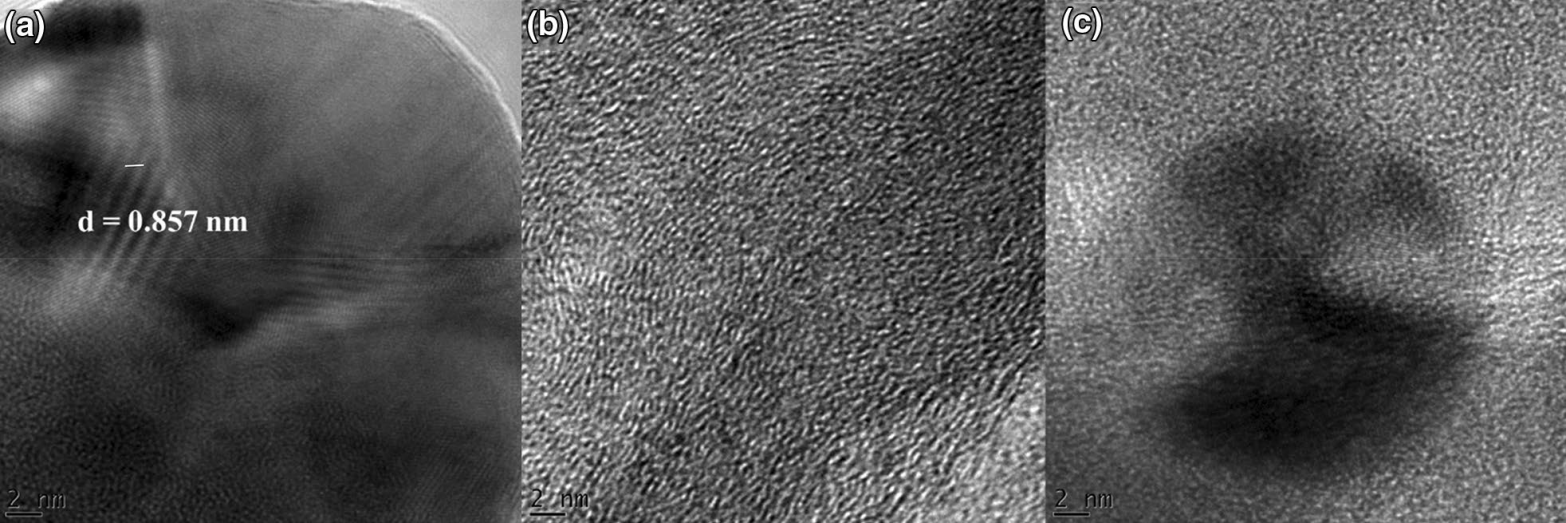
HRTEM images of (**a**) MOF, (**c**) Pd@GO, and (**e**) Pd@GO/MOF composite.

Figure 6a–e illustrates the TEM images of MOF, GO, Pd@GO, Pd@MOF, and Pd@GO/MOF, respectively, which shows a clear distinction among the texture of the samples. The image of MOF (Fig. 6a) shows the well-defined octahedral shapes of this crystalline material. After the introduction of Pd, the structure of the MOF develops rough surfaces which can be due to the interaction of Pd on the material surface (Fig. 6d)^28^. As seen in Fig. 6c for Pd@GO with respect to the TEM image of GO (Fig. 6b)^31^, the image shows well-exfoliated graphene nanosheets and wrinkled transparent sheet-like structure. In the structure of Pd@GO/MOF composites (Fig. 6e), the layers have been established as the interchange between Pd@GO sheets and MOF blocks. It was seen that the oxygen groups of GO in Pd@GO link with the metals of copper dimmers in MOF structure to form a composite^26^ with reference to the neat MOF (Fig. 6a) and Pd@MOF (Fig. 6d^28^. It is interesting to observe that again in the composite, the particles conserved the profile of MOF crystals (see high magnification image in Fig. 6f) signifying that the constrain effects caused by layers of Pd@GO resulting in maintaining the shapes of the original MOF^26^. As shown in Fig. 6g–i, the EDX spectra and Table 1 revealed all the elements present in MOF, Pd@GO, and Pd@GO/MOF confirming the incorporation of Pd@GO onto MOF, respectively. It was observed that the presence of small peaks of Na, Mn, and S are due to impurities trapped in the GO and Pd@GO/MOF during the synthesis process as seen in EDX spectra. The presence of unwanted impurities in graphene is known to have a significant impact on their structural properties^31^. Even though some of this novel HER electrocatalysts may be presented as metal-free, the traces of metallic impurities in the composites could play a role in altering their electrochemical properties for hydrogen evolution reaction^23^. In addition, Fig. 7a demonstrates that the phase contrast of the intact crystal of MOF was barely visible. The measured interplanar d spacings of 0.857 nm (8.57 Å, estimated from scale bar measurement) correspond to MOF in (222) orientation in HRTEM. In addition, the crystallinity of the MOF observed in these images shows the clear diffraction spots which are in agreement with XRD discussed above. The HRTEM image (Fig. 7b) of Pd@GO shows an amorphous characteristic of graphene sheets^24^. The Pd@GO/MOF image in Fig. 7c for HRTEM shows the spacing is 1.00 nm (10.0 Å) which is close to the d-spacing of (222) plane of MOF and (002) plane of GO. These results clearly suggest the development of the hybrid structure of Pd@GO/MOF with reference to as-prepared MOF (Fig. 7a,b).

**Figure 8:**
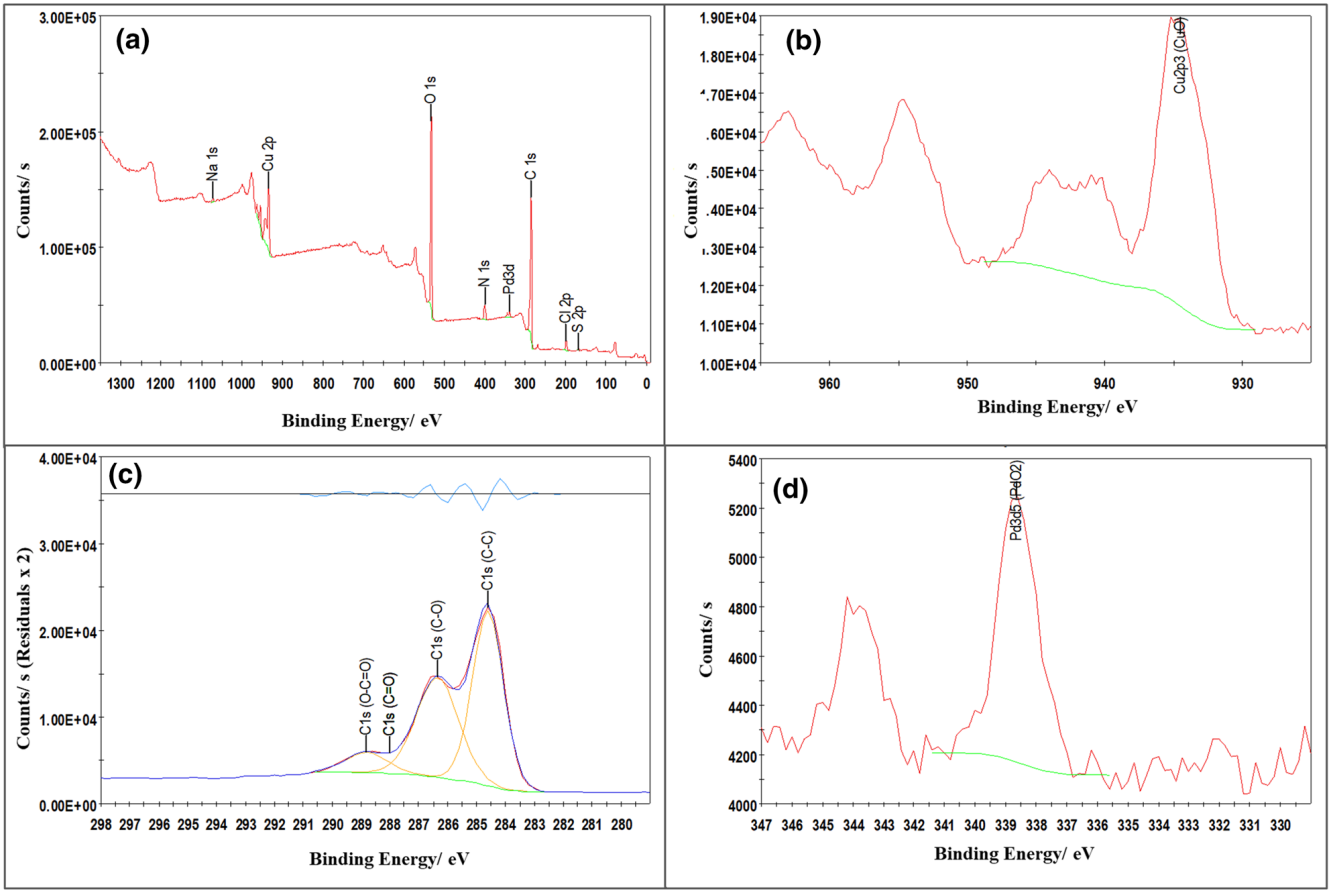
XPS of Pd@GO/MOF composite; (**a**) survey, (**b**) Cu2p, (**c**) C1s and O1s curves.

To understand the elemental composition of Pd@GO/MOF composite and its main constituents (i.e. MOF, GO, and Pd@GO), X-ray photoelectron spectroscopy (XPS) was employed as an analytical tool to identify the valence states of Pd and Cu in the composite materials and also to identify elements that exist within a material to support EDX results. The survey spectrum of Pd@GO/MOF composite in Fig. 8a clearly shows the existence of Cu^2+^ together with oxygen, carbon, nitrogen, and palladium as an indication of composite formation. Table 2 provides elemental identity and quantification of the expected elements in MOF, GO, Pd@GO, and Pd@GO/MOF materials. The presence of Pd with a 0.2 atomic percentage was observed in both Pd@GO and Pd@GO/MOF composite. The decrease in the amount of Cu from 9.6 at.% in the MOF to 3.5 at.% in the final composite was due to the incorporation of Pd@GO in the MOF structure. As indicated in Fig. 8b, the Cu2p3 satellite peak is observed at 934.8 eV binding energy and it represents the CuO present in the MOF. The C1s binding energy of the composite is observed in Fig. 8c, the three peaks observed on the C1s spectra corresponds to the C–C at ~ 284.6 eVwith 32.2 at.%, C–O at 286.4 eV with 25.2 at.% and O–C = O at 288.8 eV with 5.2 at.%. The presence of the C–C peak proves that more of the carbon atoms were put in a hexagonal structure in MOF due to the introduction of Pd@GO. The Pd3d spectrum of Pd@GO/MOF (Fig. 8d) can be due to oxidation of Pd, shown at 338.8 eV.

**Table 2:** XPS elemental ID and quantification for MOF, GO, Pd@GO, and Pd@GO/MOF.

Material	Name	Peak BE	FWHM eV	Atomic %
MOF	C1s	285	3.4	56.4
	O1s	531.9	3.2	34
	Cu2p	935.2	4.6	9.6
GO	C 1 s	287	4.2	61.8
	O1s	533.3	3.2	34.7
	N 1 s	402.2	3.7	1.8
	S 2p	169.6	3.2	1.2
	Cl 2p	201	3.4	0.5
Pd@GO	C 1 s	285.2	4.3	67.6
	O1s	532.1	3.2	27.1
	N 1 s	400.3	4.2	3.1
	Cl 2p	197.7	3.6	1.5
	S 2p	168.1	3.1	0.3
	Na 1 s	1071.3	3.6	0.3
	Pd3d	337.8	3	0.2
Pd@GO/MOF	C 1 s	285.3	4	64.1
	O 1 s	532.2	3.4	27
	N 1 s	400	3.6	3.5
	Cu 2p	934.8	4.5	3.5
	Cl 2p	198.8	3.7	1.4
	Na 1 s	1071.8	3.7	0.2
	Pd3d	338.8	2.8	0.2
	S 2p	168.3	1.2	0.1

### Electrochemical characterization

**Figure 9:**
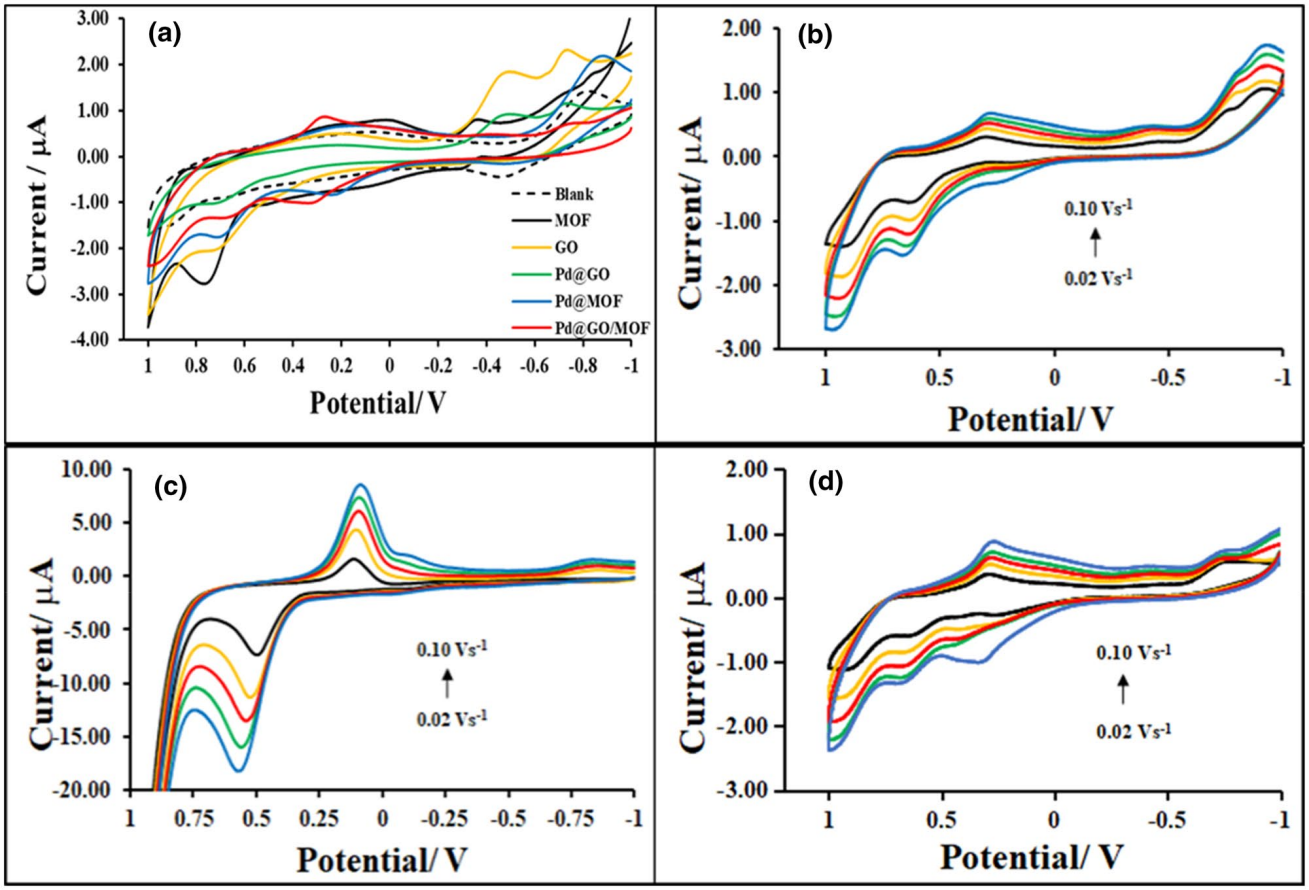
(**a**) CV curves of Blank, MOF, GO, Pd@GO, Pd@MOF and Pd@GO/MOF composite at 0.1 v s^−1^ in 0.1 M TBAP/DMSO electrolyte solution on Au electrode. (**b**–**d**) MOF, Pd@GO and Pd@GO/MOF at different scan rates (0.02–0.1 v s^−1^) in 0.1 mol L^−1^ TBAP/DMSO, respectively.

Electrochemical behaviours of MOF, GO, Pd@GO, Pd@MOF, and Pd@GO/MOF composite were investigated using Au electrode in TBAP/DMSO electrolyte (0.1 mol L^−1^) as a supporting electrolyte due to its good stability and electrical conductivity of the system. The voltammograms are represented in Fig. 9a. The oxidation and reduction process of the blank gold electrode was observed at around − 0.50 V which was similar to the one reported in the literature^36^. The voltammogram of MOF displays three oxidation processes attributed to the oxidation copper from Cu/Cu^1+^, Cu^1+^/Cu^2+^, and Cu^2+^/Cu^3+^ between 0 and 1 V^37,38^. On the other hand, the CV of Pd@MOF in the same condition shows enhancement of both oxidation and reduction peaks as compared to MOF and this clearly indicates the introduction of Pd on the MOF surface. The CV of GO material shows characteristic cathodic reduction peaks at the region of − 0.500 and − 0.750 V (vs Ag/AgCl) due to the electrochemical reduction of epoxy, peroxy, and aldehyde functional groups^31^. The quasi-reversible processes were observed in the voltammogram of Pd@GO in the same potential window due to the electroactive species of GO^39^ and Pd metal^39^ which is clearly seen in Fig. 9c. The CV of Pd@GO/MOF on the gold electrode still allows the diffusion of the redox mediator (Cu^2+^/Cu^+^) through their layers to the electrode surface^28^ with the enhancement of oxidation and reduction peaks at around 0.3 V for conversion of Cu/Cu^+^ and Cu^3+^/Cu^2+^, respectively.

**Figure 10:**
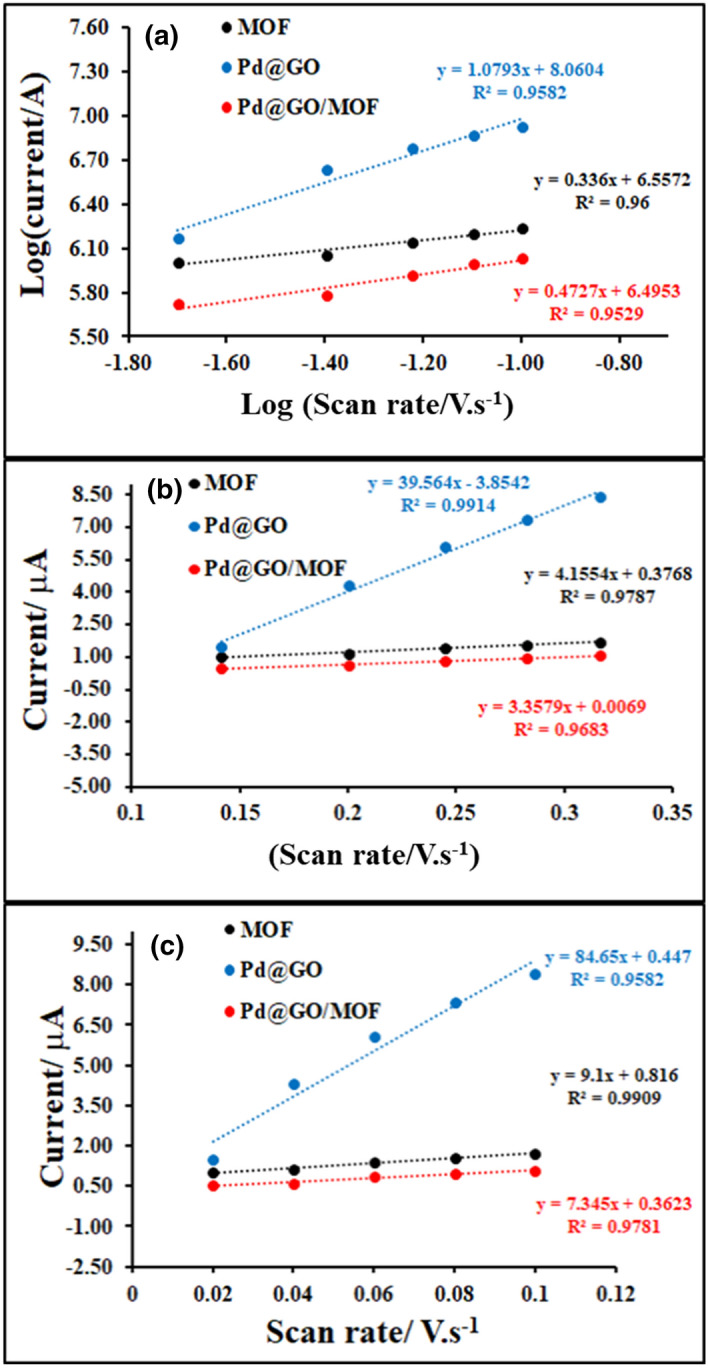
(**a**) The log–log plot of the absolute value of the peak current vs scan rate, (**b**) peak current as a function of the square root of scan rate, and (**c**) peak current as a function of scan rate for MOF, Pd@GO and Pd@GO/MOF on gold in 0.1 M DMSO/TBAP electrode system at different scan rates (0.02–0.10 V s^−1^).

The scan rate studies of MOF, Pd@GO, and Pd@GO/MOF composite were performed at various scan rates. The CV curves of MOF, Pd@GO, and Pd@GO/MOF composite are shown in parts b-d of Fig. 9, respectively. As shown in Fig. 9b for MOF, and (d) for Pd@GO/MOF, the peak currents increase with an increase in scan rate due to the generation of Cu ions which are used as catalysts to the HER mechanism^37,40^. Figure 9c demonstrates multiscan voltammograms of Pd@GO which shows well-maintained reversible peaks with the increase in scan rate. From the scan rate studies, it was observed that both anodic (*I*_pa_) and cathodic peak (*I*_pc_) currents are directly proportional to the scan rates from 0.1 to 1 V s^−1^. It was seen that the oxidation and reduction couples in MOF and Pd@GO/MOF displayed an electrochemical quasi-reversible process because of the change in peak potential (Δ*E*_p_) and *I*_pa_/*I*_pc_ ratios. However, the unity of *I*_*pa*_*/I*_*pc*_ ratios with respect to Cu^1+^/Cu^2+^ and its reverse couple; and the logarithm-logarithm of the absolute value of the reductive peak current and scan rate with the slope of 0.5 as shown in Fig. 10a and Table 3 indicate diffusion-controlled characters of the oxidation and reduction processes^41,42^. In addition, the Randles–Sevcik equation was used to determine the diffusion coefficient, *D*, for a quasi-reversible system as given in Eq. (1)^41,42^.

1 $$I_{{\text{p}}} = \left( {{2}.{65} \times {1}0^{{5}} } \right)n^{{{3}/{2}}} ACD^{{{1}/{2}}} \left( v \right)^{{{1}/{2}}}$$

**Table 3:** Electrochemical parameters of MOF, Pd@GO, Pd@MOF, and Pd@GO/MOF on gold in 0.1 M DMSO/TBAP electrode system at different scan rates (0.02–0.10 V s^−1^).

Material	*I*_pa_/*I*_*p*c_	Log(*I* vs *v*^1/2^ ) Slope	*D* (cm^2^. s^−1^ )	*Γ* (mol cm^−2^)
MOF	2.39	0.34	1.39 × 10^−7^	1.32 × 10^−10^
Pd@GO	5.73	1.08	1.08 × 10^−4^	1.27 × 10^−9^
Pd@MOF	1.65	0.33	1.90 × 10^−6^	1.69 × 10^−10^
Pd@GO/MOF	1.20	0.47	7.79 × 10^−7^	1.09 × 10^−10^

where, *n, A, D, C,* and *v* are the number of electrons transferred, electrode area in cm^2^, diffusion coefficient in cm^2^ s^−1^, the bulk molar concentration of the electroactive species in mol cm^−3^, and scan rate in V s^−1^, respectively.

Figure 10b exhibits the increase in current as a function of the square root of the scan rate, *ν*^1/2^ which is consistent with Eq. (1). The *D* values as presented in Table 1, were found to be 1.39 × 10^−7^, 4.15 × 10^−7^, 1.08 × 10^−4^, 8.8 × 10^−7^ and 7.79 × 10^−7^ cm^2^ s^−1^ for MOF, GO^31^, Pd@GO, Pd@MOF^28^ and Pd@GO/MOF, respectively. The diffusion coefficient is higher than the one of MOF; a slight increase in the *D* for Pd@GO/MOF composite is attributed to the improved electrical conductivity caused by the presence of GO in the framework. The decrease in *D* for the MOF material indicates the repulsive nature of MOF^28^. The introduction of Pd@GO on the MOF surface enhanced the diffusion coefficient of the composite compared to the parent MOF. A similar trend was observed in MOF-based polymer composite^21,22^.

Furthermore, it was seen that instead of the material undergoing only a diffusional process, it can also adsorb on the surface of the electrode^43^. This behaviour can be obtained by directly relating the peak current with the surface coverage (Γ) and the scan rate, where *F, R,* and *T* are Fardaic constant, gas constant, and temperature as presented in Eq. (2).

$$I_{{\text{p}}} = \frac{{n^{2} F^{2} \Gamma Av}}{4RT}$$

Figure 10c shows a linear association of current and scan rate where regression line equation and R^2^ are presented to determine the surface coverages (Table 3) for MOF, Pd@GO, and Pd@GO/MOF, and their values were found to be 1.32 × 10^−10^, 1.27 × 10^−9^, and 1.09 × 10^−10^ mol cm^−2^, respectively, confirming the adsorption of the material on the gold electrode.

### Hydrogen studies

**Figure 11:**
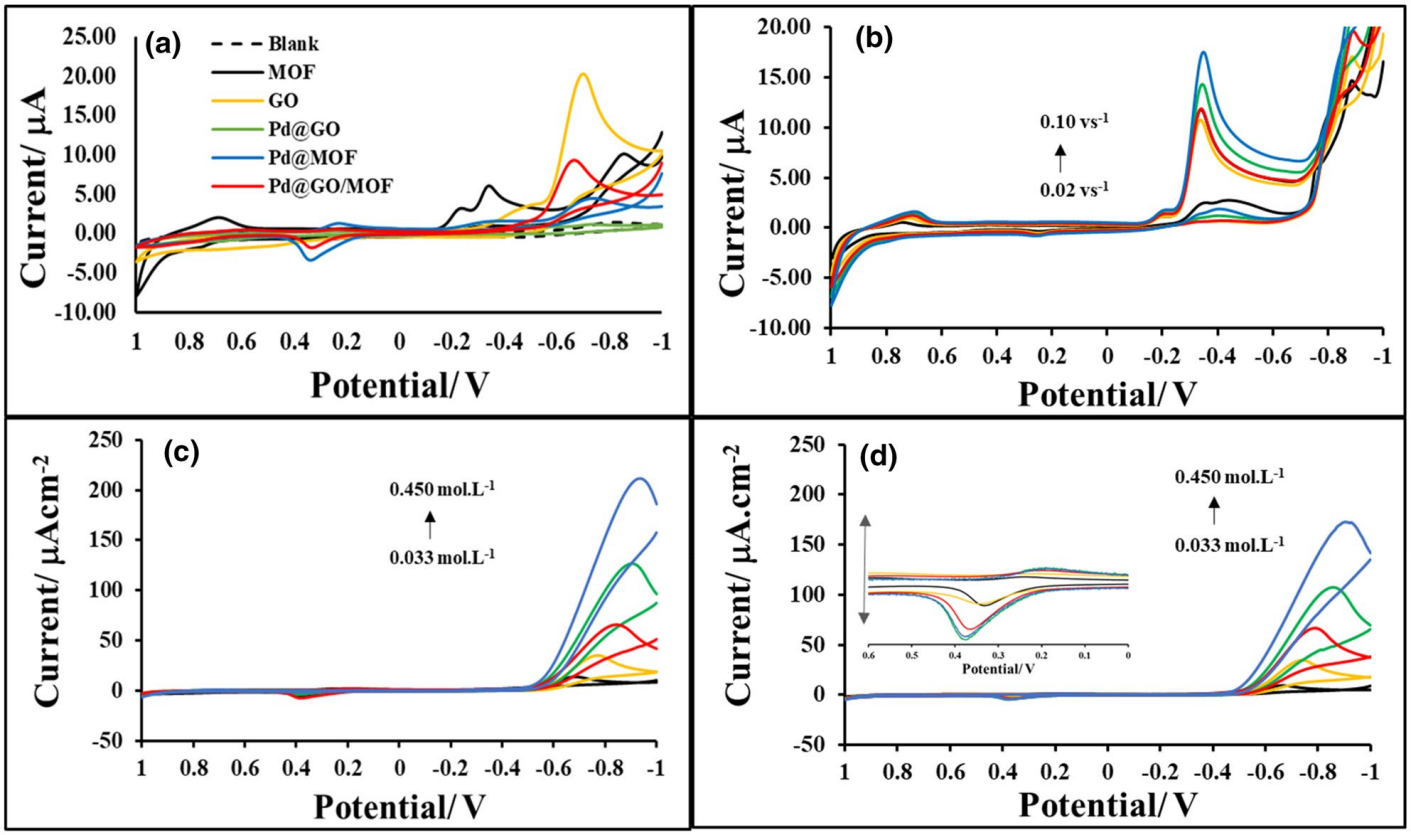
(**a**) CV curves of Blank, MOF, GO, Pd@GO, Pd@MOF and Pd@GO/MOF composite in the presence of 0.033 mol L^−1^ H_2_SO_4_ at 0.10 V s^−1^ and CV curves of (**b**) MOF, (**c**) Pd@MOF and (**d**) Pd@GO/MOF composite in different concentration of hydrogen source (0.033–0.450 mol L^−1^ H_2_SO_4_; Inset: CV enlargement at the selected area) at 0.10 V s^−1^ on Au electrode in 0.1 mol L^−1^ TBAP/DMSO electrolyte solution.

The electrochemical hydrogen activities of all prepared samples were measured using H_2_SO_4_ as a proton source in 0.1 M TBAP/ DMSO electrolyte using a three-electrode system. Figure 11a presents the plot of current vs potential of the blank gold electrode, MOF, Pd@GO, and Pd@GO/MOF as compared to GO^31^ and Pd@MOF^28^. As compared to CV plots in Fig. 11a in absence of a proton source, upon addition of H_2_SO_4_, a new cathodic wave on MOF, GO, Pd@MOF, and Pd@GO/MOF appeared at an onset potential of − 0.5 V with an increase in cathodic current. However, this behaviour was not observed in the case of Pd@GO, which designates that there was no hydrogen evolution. From this observation, it can be deduced that the MOF, GO, Pd@MOF and Pd@GO/MOF can reduce the hydrogen protons (H^+^) to form molecular hydrogen (H_2_) at lower potentials^22^. In order to substantiate that the current obtained is a source of the cathodic wave, the concentration-dependent studies for MOF, Pd@MOFand Pd@GO/MOF were carried out and the results are presented in Fig. 11b–d, respectively. Moreover, it can be seen that an increase in H_2_SO_4_ concentration results in a further increase in current reading at lower onset potential, thus the new catalytic wave is due to the proton source. Furthermore, a small anodic peak in Fig. 11c, d (see enlargement in Fig. 11d) is attributed to the molecular hydrogen adsorption by the material as observed by Monama et al*.*^28^. The adsorption of produced molecular hydrogen by the material was due to the presence of Pd particles as an indicator of the improved hydrogen spillover mechanism^28^ and also the formation of Pd hydride^41,44^.

The Tafel plot was constructed from current density-potential data at various concentrations ranging from 0.033 to 0.450 mol L^−1^ H_2_SO_4_ for MOF, and Pd@GO/MOF nanocomposite whereby the current, *i,* is measured as a function of overpotential, η. Ramohlola et al.^20,22^, reported that the Tafel slope, *b,* could also serve as an indicator of either Volmer, Heyrovsky, and Tafel in a multi-step proton transfer process while *i*_0_ measures the performance of an electrocatalyst. In this study, the values of *b* and *i*_0_ were estimated by linear polarization curves and the results are given in Table 4. In addition, another important parameter that can give insights into the reaction mechanism is the cathodic transfer coefficient (1-α) which can be estimated using the Tafel equation (Eq. 3).

3 $$b = \frac{ - 2.303RT}{{(1 - \alpha )F}}$$

**Table 4:** Experimental values of Tafel slope (*b*), charge transfer coefficient (1–*α*)*,* exchange current density (*i*_0_), and TOF of *MOF and Pd@GO/MOF* composite.

Material	H_2_SO_4_ (mol L^**−**1^)	Slope (*b*) (V dec^**−**1^)	-*b* (mV dec^**−**1^)	1-*α*	log*i*_*0*_ (μA m^**−**2^)	*i*_*0*_ (A m^**−**2^)	*TOF* (mol H_2_ s^**−**1^_)_
MOF	0.033	− 0.2544	254.4	0.23	6.28	1.9	0.61
	0.075	− 0.1883	188.3	0.31	6.61	4.1	1.30
	0.150	− 0.1763	176.3	0.34	6.9	7.9	2.53
	0.300	− 0.1708	170.8	0.35	7.1	12.6	4.01
	0.450	− 0.1551	155.1	0.38	7.2	15.8	5.05
Pd@GO/MOF	0.033	− 0.3000	300.0	0.20	6.39	2.5	0.78
	0.075	− 0.2284	228.4	0.26	6.66	4.6	1.46
	0.150	− 0.1958	195.8	0.30	6.97	9.3	2.97
	0.300	− 0.1699	169.9	0.35	7.17	14.8	4.71
	0.450	− 0.1576	157.6	0.38	7.39	24.5	7.82
GO^31^	0.300	− 0.1440	144.0	0.41	7.15	14.1	2.93
Pd@GO	0.300	− 0.1927	192.7	0.31	6.90	7.10	3.11
Pd@MOF^28^	0.300	− 0.1930	193.0	0.31	6.9	7.1	2.26
Pd@CuPc/MOF^28^	0.300	− 0.1770	177.0	0.33	7.0	8.9	2.83
GO/MOF^31^	0.300	− 0.1250	125	0.47	7.24	17.4	4.57
FeSe_2_/GO^45^	0.500	− 0.0640	64	–	–	–	–
WS2/rGO^46^	0.500	− 0.0520	52	–	–	–	–
Ni/NiO@rGO^12^	0.500	− 0.0630	63	–	–	–	–

**Figure 12:**
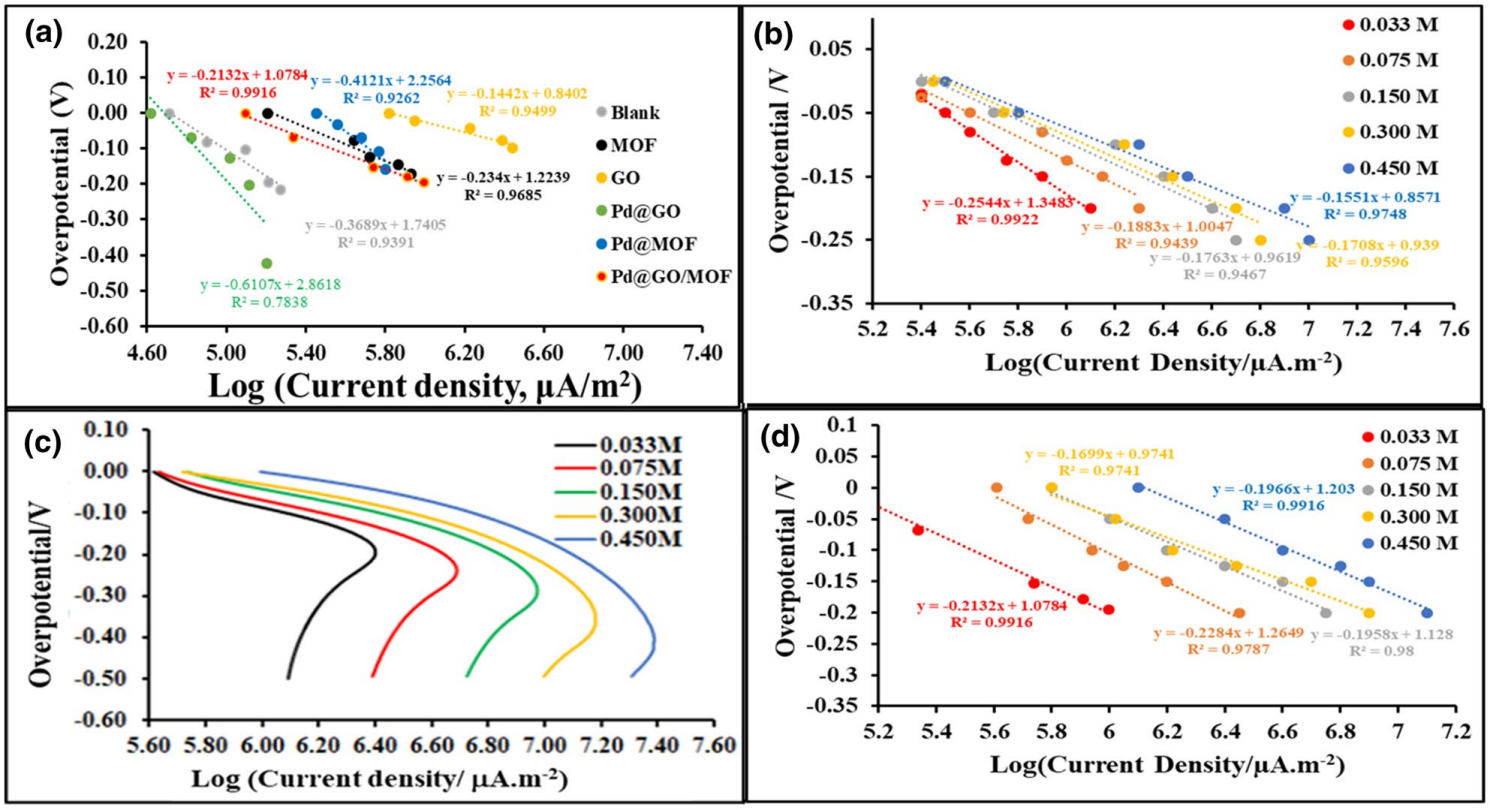
Tafel plots of (**a**) blank, MOF, GO, Pd@GO, Pd@MOF and Pd@GO/MOF composite (~ 2.0 × 10^−4^ mol L^−1^) in the presence of 0.033 mol L^−1^ H_2_SO_4_ at 0.10 V s^−1^. (**b**) Tafel plots of MOF; (**c**) polarisation curve and (d) Tafel plots of Pd@GO/MOF composite in different concentrations of H_2_SO_4_ and 0.10 Vs^−1^ scan rate on Au electrode in 0.1 mol L^−1^ TBAP/DMSO electrolyte solution.

**Scheme 1:**
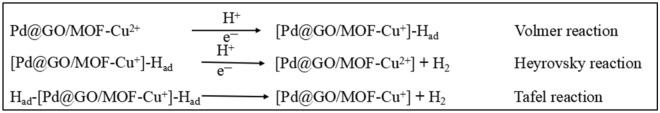
Proposed mechanisms involved in the HER kinetics of the nanocomposite.

Figure 12 and Table 4 show Tafel plots and parameters of Au electrode, MOF, and Pd@GO/MOF composite. It was seen that the blank electrode has a Tafel slope of 200.8 mV dec^−1^ at 0.300 mol L^−1^ H_2_SO_4_. At the same acid conditions, MOF and Pd@GO/MOF composite showed lower Tafel slope values as compared to the GO^31^ and Pd@MOF^28^. The Tafel slopes of the composite were observed to between 169.9 and 158.1 mV dec^−1^ at 3.00 and 4.50 mol L^−1^, respectively. This suggests that the HER on the prepared electrocatalysts proceeds via Volmer mechanisms, with the adsorption of the proton on the electrocatalyst surface (Volmer step) as the rate-determining step (Scheme 1). It was reported that the Tafel slopes of 105–150 mV dec^−1^ in HER describes the Volmer mechanism as a rate-determining method^8^. The results are in good consent with the work reported by Kubisztal et al*.*^47^ when studying the HER behaviour of nickel-based molybdenum composite. It was also shown that a Tafel slope of more than 120 mV dec^−1^, originates from either the Volmer rate-determining step or the Heyrovsky rate-determining step with high adsorbed hydrogen atom coverage^48^.

Moreover, the charge-transfer coefficient (α) of 0.5 is known to describe the Volmer reaction or the Volmer reaction together with Heyrovsky or Tafel reactions^8^. As given in Table 4, the α values of MOF and Pd@GO/MOF composite are approaching 0.5 confirming the proposed Volmer mechanism as a limiting step^8,47,49^. It was seen that GO/MOF composite exhibited charge transfer coefficients that are close to 0.5, showing that the rate-determining steps as Volmer coupled with Tafel or Heyrovsky mechanism^31^. Digraskar and co-workers^50^ reported MoS_2_-rGO coupled with Cu_2_ZnSnS_4_ (CZTS) nanoparticles (CZTS/MoS_2_-rGO) which exhibited the highest HER activity with an overpotential of 50 mV vs. RHE at 10 mA cm^−2^ and a Tafel slope of 68 mV dec^−1^ as an indication of the Volmer-Heyrovsky mechanism. In addition, Sathe et al*.*^51^ obtained HER Tafel slopes of ∼99 mV dec^−1^at current densities of 1.4 × 10^−3^ mA cm^−2^ for B-substituted graphene oxide in 0.5 M H_2_SO_4_ solution. On the other hand, the exchange current density (*i*_0_) was estimated from the extrapolation of the Tafel curves^20,21^. The exchange current densities obtained for different electrocatalysts are recorded in Table 4 and these values increase with an increase in H_2_SO_4_ concentration. The obtained exchange current densities are higher compared to those reported in other studies^8,17,45–47,52^. From this, it can be concluded that the prepared electrocatalyst possesses fast electron transfer, a large surface area, and favourable HER activities^9^.

**Figure 13:**
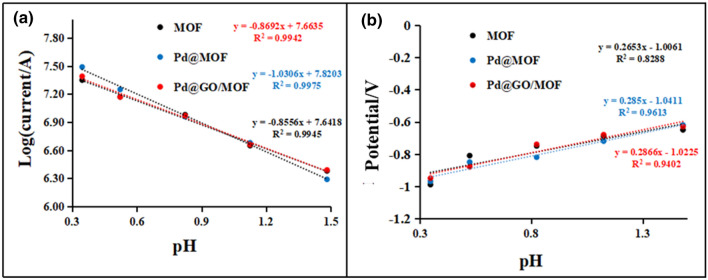
(**a**) Plot of log current as a function of pH of the solution and (**b**) Pourbaix diagram of hydrogen evolution reaction for MOF, Pd@MOF, and Pd@GO/MOF.

In addition, the turnover frequency (TOF) which is defined as the number of hydrogen molecules evolved on an active site in a time period, is also employed to evaluate the catalytic efficiency of the materials^31^. TOF quantifies the specific activity of catalytic centres and can be used to compare various materials^53^. Equation 4 presents the method to determine the TOF values of the material^53^:

4 $${\text{TOF}} = \frac{jM}{{2Fm}}$$

where j denotes current density, M is the mass percentage of prepared materials, F defines the Faraday's constant, whereas m represents the mass per square centimetre of electrocatalysts estimated from BET surface area of MOF (614. 7 m^2^/g^28^) and GO (423 m^2^/g^54^). The TOF values for MOF, Pd@MOF, and Pd@GO/MOF composite are presented in Table 4. It was seen that Pd@GO/MOF synthesised materials resulted in the enhancement of the TOF value as the concentration of H_2_SO_4_ increases, for example, the value was obtained to be 7.8 mol H_2_ s^−1^ at the highest H_2_SO_4_ concentration (0.45 mol L^−1^). The analysis of the TOF data provides information for unraveling the intrinsic activity of electrocatalysts, as shown in Table 4, Pd@GO/MOF exhibits a slightly higher TOF value of 4.7 mol H_2_ s^−1^ than that for GO, MOF, and Pd@MOF under similar conditions of 0.300 mol L^−1^ H_2_SO_4_. It is worth mentioning that Pd@GO/MOF composite also showed a higher hydrogen generation rate of 7.8 mol H_2_ s^−1^ than MOF at 0.450 mol L^−1^ H_2_SO_4_ and makes it to be the best HER electrocatalyst in acidic medium. Moreover, Fig. 13a, b present the pH dependence of the current density and potential, respectively, for the experimental data obtained in the present study for MOF, Pd@MOF, and Pd@GO/MOF. The slope of the trendline in Fig. 12a (log(*i*) vs. pH), signifies the HER reaction order which was determined to be ~ 0.8, suggesting a mixed type mechanism of the Volmer coupled with Heyrovsky or Tafel steps^55^ as observed in Tafel parameters. Furthermore, Fig. 13b shows a representation of the Pourbaix diagram, and the slope was seen to be less than 0.5. The observed dependence agrees well with the proposed mechanism of the process above.

**Figure 14:**
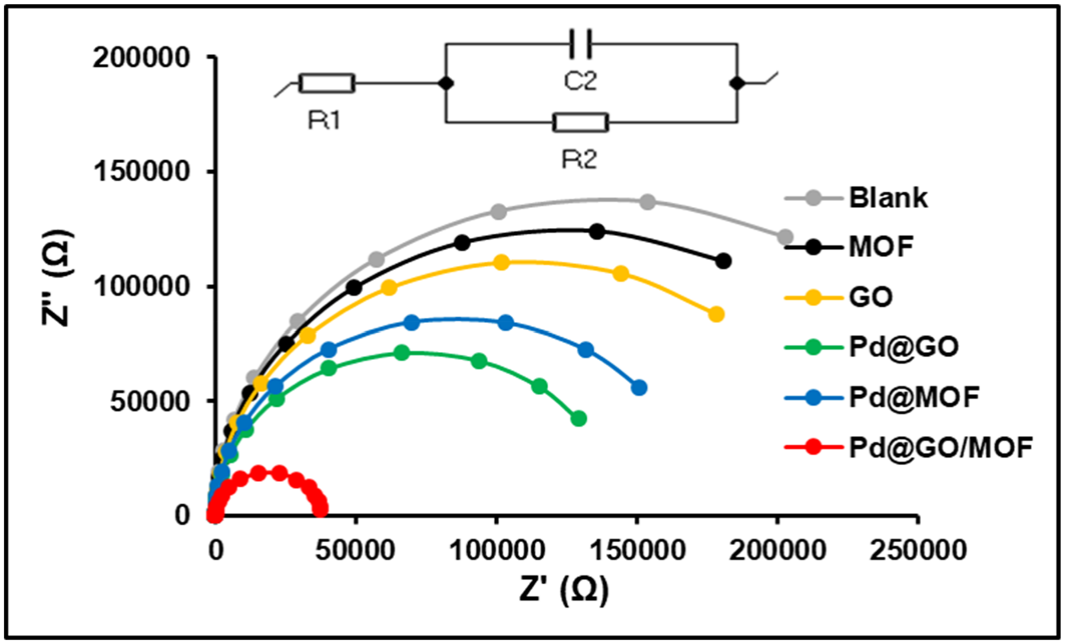
Nyquist plots of the Blank, MOF, GO, Pd@GO, Pd@MOF, and Pd@GO/MOF composite in 0.5 mol L^−1^ H_2_SO_4_. electrolyte at OCP with the frequency range 5.0 × 10^5^–0.7 Hz.

Electrochemical impedance spectroscopy (EIS) is one of the electrochemical characterisation techniques that can reveal the electrical resistance of several materials. It is an important technique to disclose the conventional electrochemical measurements, interfacial interaction and to study the electrochemical kinetics of the HER process^56,57^. Figure 14 shows the overlaid Nyquist plots of a blank, MOF, GO, Pd@GO, Pd@MOF, and Pd@GO/MOF composite in 0.5 mol L^−1^ H_2_SO_4_ as a supporting electrolyte and hydrogen source under open circuit potential (OCP) with the frequency range 5 × 105–0.7 Hz. The Nyquist plots were fitted with the electrical equivalent circuit (EEC), which encompasses Voigt RC elements, involving a solution/ electrolyte resistance (Rs indicated as R1), charge-transfer resistance (Rct signified as R2), and constant phase elements (CPE or Q) as shown by the inset of Fig. 14. It was known that the diameter of the semicircle of the Nyquist plot gives information about charge transfer resistance (Rct) of the interface, and its value is obtained by fitting the EEC^55^. As seen in Fig. 14, the semicircles for all materials are smaller than blank, which could be the reason for enhanced electrochemical properties. It was reported in the literature that the radius of high-frequency semicircle divulges hydrogen absorption and low-frequency semicircle represents the HER kinetics^56^. As observed in Fig. 14, the Rct for Pd@GO/MOF composite is smaller than the MOF value as shown in Table 5 (i.e., from 249 to 37.7 kΩ), indicating the excellent conductivity of the composites due to the presence of Pd@GO. Rct of materials follows the sequence of blank < MOF < GO < Pd@MOF, Pd@GO < Pd@GO/MOF. Accordingly, a small semicircle describes a high HER activity with a small Rct value for the Pd@GO/MOF composite. In addition, EIS results display a reduced charge transfer resistance and improved conductivity because of the presence of graphene oxide support. In contrast, the constant phase element (CPE or C2) was given to evaluate the exposed active surface area of the electrode. As shown in Table 5, MOF exhibits the largest CPE whereas composite has a 0.457 µF CPE value. Consequently, results advocate that the synthesised Pd@GO/MOF composite is a good candidate for hydrogen evolution reaction.

**Table 5:** Fitted circuit parameters of electrochemical impedance spectroscopy (EIS) spectra for the blank, MOF, GO, Pd@GO, Pd@MOF, and Pd@GO/MOF composite in 0.5 mol L^−1^ H_2_SO_4_.

Material	R_*s*_ (Ω)	CPE µF	R_*ct*_ (kΩ)
Blank	9.32	0.493	276
MOF	8.98	0.560	249
GO	10.1	0.404	222
Pd@GO	12.3	0.521	143
Pd@MOF	10.3	0.490	172
Pd@GO/MOF	11.3	0.457	37.7

## Conclusions

The Pd@GO/MOF nanocomposite as an efficient electrocatalyst was successfully prepared from MOF and Pd@GO via impregnation procedure. The presence of GO was very important for the support of Pd atoms through the diffusion process to form Pd@GO/MOF and the addition of defects into GO to expose catalytic sites. The influences of Pd@GO on the structural properties of MOF surface, as well as electrochemical activities for hydrogen evolution reaction, were systematically investigated. The observed promotional roles of incorporated Pd@GO on MOF surface are endorsed to possible synergetic effects between Pd and GO with MOF matrix, resulting in an improvement of HER as part of hydrogen production with enhanced TOF values. Moreover, the b and α values displayed that the HER rate-determining step on the studied MOF and Pd@GO/MOF nanocomposite may be the Volmer mechanism or the Volmer mechanism together with one of other two reactions supported by slopes from the plot of log current vs pH and Pourbaix diagram. In this work, the reported strategy of Pd@GO/MOF nanocomposite which exposes the catalytic active sites of the MOF core, while, maintaining the porous carbon skeleton of GO and the presence of Pd nanoparticles, posed a great potential for more applications in hydrogen production and storage technology thorough hydrogen evolution reaction.
